# Tau conformation, distribution and PET imaging correlations in progressive supranuclear palsy

**DOI:** 10.1186/s40035-026-00545-5

**Published:** 2026-03-25

**Authors:** Chong Dong, Jing-Hong Ma, Hong-Wen Qiao, Gilles D. Tamagnan, Piu Chan, Shu-Ying Liu

**Affiliations:** 1 Department of Neurology, Xuanwu Hospital, Capital Medical University, Beijing, China; 2 Department of Radiology and Nuclear Medicine, Xuanwu Hospital, Capital Medical University, Beijing, China; 3 XingImaging LLC, 760 Temple Street, New Haven, Connecticut 06510 USA; 4 National Clinical Research Center for Geriatric Diseases, Beijing, China

**Keywords:** Tau, Progressive supranuclear palsy, Microstructure, Positron emission tomography, Tracers

## Abstract

Progressive supranuclear palsy (PSP) is a primary tauopathy characterized by aggregation of pathological tau. Recent advances in cryo-electron microscopy have enabled the classification of tauopathies at near-atomic resolution, revealing disease-specific tau filament conformations. These microstructural differences may influence the intracellular localization, intercellular propagation, and spatial distribution of tau pathology, as well as the microscopic binding profiles and macroscopic imaging signatures of tau positron emission tomography (PET) tracers. This review focuses on PSP by delineating its specific tau architecture and cellular and spatial distributions and how they differ in comparison with other major tauopathies and by critically discussing the clinical utility and limitations of tau PET. Through this integrative perspective, we aim to bridge neuropathological insights with in vivo PET findings.

## Introduction

Progressive supranuclear palsy (PSP) is an adult-onset neurodegenerative disease and is an atypical type of parkinsonism. Characterized by supranuclear ophthalmoplegia and postural instability, PSP encompasses a spectrum of clinical manifestations involving motor, speech, behavioral, and cognitive abnormalities [1, 2]. Due to its heterogeneous clinical manifestations, the Movement Disorder Society has established the clinical diagnostic criteria for PSP [3], which broadly categorize PSP into distinct subtypes, including PSP-Richardson’s syndrome (PSP-RS), subcortical variants (e.g., PSP-parkinsonism [PSP-P] and PSP-progressive freezing gait [PSP-PGF]), and cortical variants (PSP-corticobasal syndrome [PSP-CBS], PSP-speech and language disorder, and PSP-frontal syndrome [PSP-F]) [4]. The clinical diversity of PSP poses significant challenges for differentiating it from other neurodegenerative diseases with overlapping motor or cognitive phenotypes.

Pathologically, PSP is associated with tau aggregation in neurons and glial cells, resulting in the formation of neurofibrillary tangles (NFTs) and neuropil threads [5]. Studies on tau distribution patterns in PSP have revealed a region-specific, hierarchical progression pattern, with core deposition along the Pallido–Nigro–Luysian axis (pallidum, substantia nigra, and subthalamic nucleus of Luys). This pathology propagates rostrally to the cerebral cortex and caudally to the cerebellum/dentate nucleus [6, 7]. From the microstructural perspective, recent cryo-electron microscopy (cryo-EM) investigations have revealed a unique three-layered fold in PSP tau filaments, a signature that differentiates PSP from other tauopathies [8]. These findings not only complement clinical diagnosis and neuropathology but also provide mechanistic insights into tauopathy pathogenesis at the molecular level.

Tau filament folding is a disease-specific, conserved conformation within the same tauopathy but is distinct across different disease entities [9]. The tau tracers used in positron emission tomography (PET) exhibit differential binding sites and affinities in different tauopathies, which may shape the varying levels of consistency between in vivo tau imaging and in vitro tau pathology. Tau tracers require excellent physical and chemical properties to penetrate the blood–brain barrier. The evolution of tau PET tracers from first- to second-generation compounds has addressed limitations such as off-target binding, low affinity for PSP-related tau isoforms, and metabolic inactivation [10].

In this review, we first focus on the microstructural discrepancies between PSP and other tauopathies, and the binding characteristics of second-generation tau tracers (^18^F-PI-2620, ^18^F-APN-1607) with PSP tau filaments at the microscopic level. Next, we review key mechanistic considerations, including tau seeding activity, disease-specific tau propagation, and cellular/temporal-spatial tau distribution [11]. Furthermore, we discuss whether tau PET tracers can reveal the distribution patterns of tau and the discriminative abilities of these tracers in differentiating PSP from other tauopathies or among variant PSP subtypes in vivo.

## Composition of the tau protein and hierarchical tau structure

**Fig. 1:**
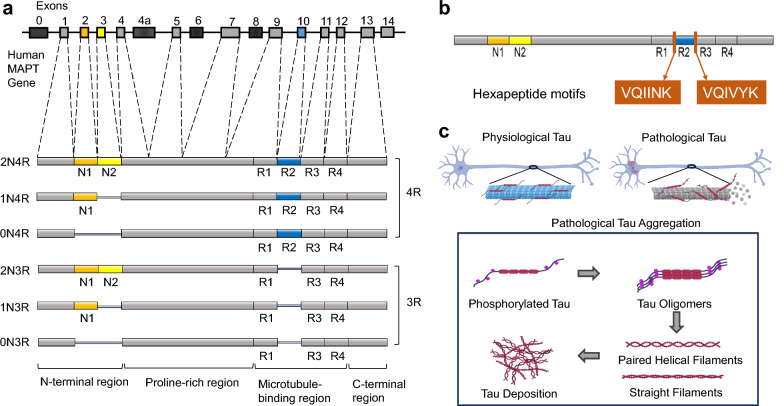
Human tau isoforms, key motifs mediating tau–tau interactions, and the process of tau aggregation. **a** Alternative splicing of the human *MAPT* gene generates six tau isoforms. These isoforms differ in the presence of 0, 1, or 2 N-terminal inserts encoded by exons 2 and 3, as well as in the inclusion or exclusion of exon 10, which produces either three or four microtubule-binding repeat domains. **b** Using the 2N4R tau isoform as an example, the hexapeptide motifs VQIINK and VQIVYK located at the start of the R2 and R3 repeat domains are highlighted, which are responsible for promoting tau–tau interactions. **c** Under physiological conditions, tau stabilizes microtubules through its microtubule-binding repeats. Under pathological conditions, however, tau dissociates from microtubules and undergoes oligomerization, forming protofibrils that further assemble into paired helical filaments (PHFs) and straight filaments (SFs), ultimately leading to mature tau deposition. The figure was created with BioRender.com

*MAPT*, the gene encoding microtubule-associated protein tau, is located on the long arm of human chromosome 17 (17q21.31). Under physiological conditions, this gene encodes soluble tau protein. The mRNA of *MAPT* subsequently undergoes alternative RNA splicing to generate six tau isoforms. These isoforms differ by the presence or absence of three alternatively spliced exons, namely, exons 2 and 3, which encode N-terminal inserts, and exon 10, which encodes the second microtubule-binding repeat domain (R2) within the microtubule-binding region (MTBR) (Fig. 1a) [12].

The structural architecture of tau comprises four distinct domains: the N-terminal region, the proline-rich region, the MTBR, and the C-terminal region [13, 14]. The MTBR is composed of four tandem repeat domains designated R1–R4. The inclusion or exclusion of exon 10 determines whether the MTBR contains three (3R) or four (4R) repeats, resulting in the formation of the 3R-tau and 4R-tau isoforms, respectively. In addition, alternative splicing of exons 2 and 3 yields 0N, 1N, or 2N N-terminal inserts. Consequently, six tau isoforms are produced, ranging from 352 amino acids (the 0N3R isoform) to 441 amino acids (the 2N4R isoform) (Fig. 1a) [14].

A feature of the primary structure of tau is that the vast majority of its amino acid sequence exists in an intrinsically disordered state, with only small discrete fragments capable of adopting transient secondary structures. Specifically, six segments within the tau sequence tend to form β-sheet structures, three fragments tend to form polyproline helices, and both the N-terminal and C-terminal regions can transiently adopt α-helical conformations [14–16]. The constitution of the tertiary tau structure is generated by self-interactions, a process that ultimately results in the formation of paperclip-shaped tau monomers [17]. Posttranslational modifications (PTMs) of tau isoforms, including hyperphosphorylation, acetylation, and truncation, are associated with tau deposition. Tau aggregation involves the hierarchical assembly of monomeric tau into oligomers, higher-order polymers, and, eventually, filamentous structures [16]. During the formation of tau filaments, two hexapeptide motifs within the MTBR, VQIINK in the R2 domain and VQIVYK in the R3 domain, play essential roles in mediating tau–tau interactions (Fig. 1b, c) [18].

Morphologically, tau filaments can be classified into two main types: paired helical filaments (PHFs) and straight filaments (SFs). The core of tau filaments is composed of a cross-β/β-helical structure, which is formed by two identical C-shaped protofilaments. In the case of SFs, the arrangement of the two protofilaments runs parallel to each other, whereas in PHFs, they exhibit a helical twisting pattern [9, 19]. On the basis of the predominance of 3R- and 4R-tau isoform composition within tau filaments, tauopathies are generally divided into three distinct categories: 3R tauopathies, 4R tauopathies, and 3R/4R tauopathies [13].

## Core structure differences at the near-atomic level of different tauopathies

The methodologies for detecting specific tau structures have evolved over time. Electron microscopy offers unique advantages over optical microscopy, revealing that tau filaments consist of a core structure formed by the MTBR and C-terminal domains, surrounded by a “fuzzy coat” composed of the N-terminal region, the proline-rich region, PTMs, and occasionally, other proteins or cofactors. The paired stacking of tau molecules extends into either twisted or parallel arrangements within the filaments [20]. In the past decade, the development of cryo-EM has made it possible to resolve tau filament structures at near-atomic resolution [19]. To date, at least 15 distinct tau conformations have been resolved, which has prompted the proposal of classification frameworks for tauopathies on the basis of near-atomic tau conformations. These emerging frameworks highlight the heterogeneity of tauopathies [8], particularly in the core structures of tau filaments. The common components of the core structures are generally composed of R3 and R4 and 10–13 amino acids at the C-terminals.

In 3R/4R tauopathies, the R1 or R2 domain contributes only 1–2 amino acids to the core structure. According to the tau configuration, 3R/4R tauopathies can be divided into two major categories. The first is tau folds observed in Alzheimer’s disease (the AD-fold), which are also observed in both primary age-related tauopathy and familial dementia. Despite the divergent clinical manifestations, the tau filaments of these tauopathies belong to the same structural category with a C-shaped architecture [8]. The second category is tau folds observed in a significant proportion of chronic traumatic encephalopathy. These tau folds are characterized by a more open, J-shaped architecture [21, 22].

**Fig. 2:**
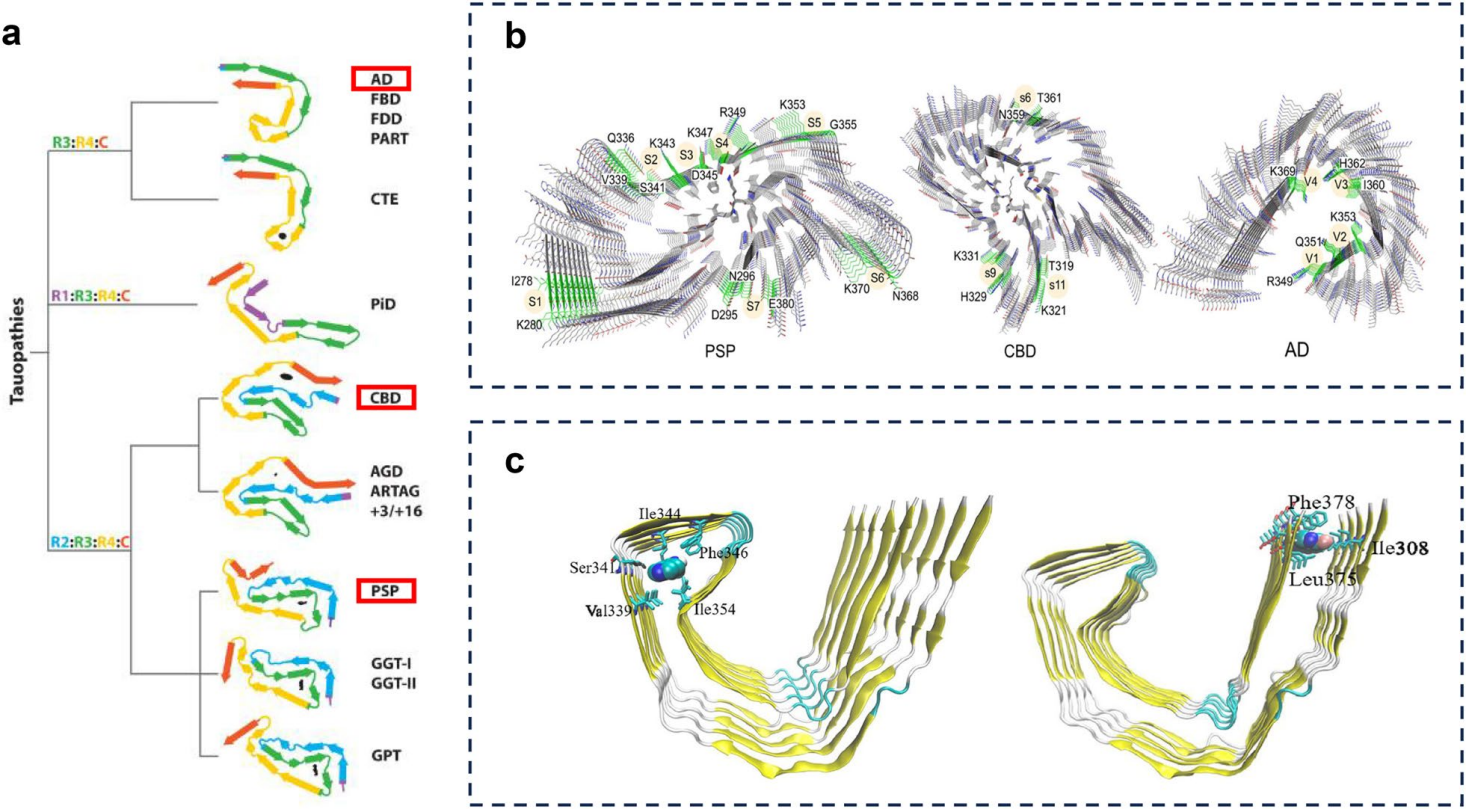
Near-atomic structures of major tauopathies and binding sites of tracers with tau filaments. **a** Near-atomic structural cores of representative tauopathies identified. In Alzheimer’s disease (AD), tau filaments fold into C-type protofilaments, whereas corticobasal degeneration (CBD) and progressive supranuclear palsy (PSP) exhibit four-layered and three-layered filament structures, respectively—two distinct tau filament architectures specific to 4R-tauopathies. **b** Key surface binding sites of tau tracers of PSP, CBD, and AD tau filaments identified via multiple molecular simulation methods. Notably, AD tau filaments form a groove that facilitates binding with tracers. **c** Schematic illustration of the core and entry sites for tau tracers in AD. These sites exhibit higher affinity for tau tracers, with the entry site being more readily accessible and thus being considered the primary binding site in AD. Additionally, similar sites exist in PSP and CBD but are less accessible. Figure 2a, b, and c are adapted from References 8, 34, and 32, respectively, with copyright permission

The tau folds in Pick’s disease are currently the only known filament structure of 3R tauopathies. The core comprises more than half of the R1 domain and forms a single protofilament with an elongated conformation (Fig. 2a) [23]. The core of 4R tauopathies contain the entire R2 domain and two residues from the R1 domain. The specific R2 domain is associated with their conformational differences from those of other tauopathies [8, 9]. PSP and corticobasal degeneration (CBD) represent two subclasses of 4R-tau structures. They have distinct folds with three and four layers, respectively. The folds of PSP and globular glial tauopathy are characterized by a three-layered core region, where the R2 and R4 repeats stack on either side of the R3 domain to form a three-layered zigzag structure. The turning points are located at the conserved PGGG motif. In contrast, the folds of CBD and argyrophilic grain disease involve stacking of the C-terminal domain with a portion of the R2 domain to form a fourth layer (Fig. 2a) [8].

The four-layer fold has a cavity with a relatively high density of positively charged nonprotein substances, whereas the three-layered fold features a larger cavity with a greater abundance of positive charges [8, 24]. Therefore, despite overlapping clinical features, PSP and CBD exhibit distinct filament architectures, referred to as the “PSP-fold” and “CBD-fold”, respectively [25]. Notably, the classification of 4R tauopathies on the basis of tau filament fold characteristics is consistent with the observed PTM patterns, which are similar between PSP and globular glial tauopathy but markedly different between PSP and CBD [26].

In 4R tauopathies, tau bands at 64 and 68 kDa correspond to full-length 4R tau. Additionally, full-length tau may undergo cleavage within the fuzzy coat region, yielding 33 kDa and 37 kDa bands that reflect N-terminally truncated tau species. The size difference between the 33 kDa and 37 kDa bands is hypothesized to arise from divergent arrangements at the termini of the structured cores. Specifically, on immunoblots of sarkosyl-insoluble brain extracts, the low molecular weight tau fragments of PSP are predominated by the 33 kDa tau fragment, whereas CBD is characterized by two closely related bands of approximately 37 kDa [27, 28].

## Tau tracer binding with the tau core structure

Tau PET tracers exhibit high-affinity binding sites with tau filaments [29]. Using cryo-EM, researchers have identified two major binding sites for tau tracers within the β-helical regions of PHFs and SFs in AD, along with a third major site in the C-shaped cavity of SFs [30, 31]. By applying a multiscale simulation workflow, including molecular docking, molecular dynamics simulation, metadynamics simulation, and free energy calculation, scientists have been able to refine the binding sites identified by cryo-EM and uncover additional high-affinity sites in tau fibrils in AD for tau tracers. Based on the cryo-EM structure of tau fibrils, Kuang et al. found eight surface binding sites (Fig. 2b), three core binding sites and one entry site within AD tau fibrils for the PET tracer PI2620 (Fig. 2c). Due to stronger hydrophobic interactions and less solvent exposure, the binding at the core and entry sites is more favorable than the binding at surface sites. Critically, the entry site is considered the primary binding site for tau tracers due to its easy accessibility [32].

Using this multiscale simulation workflow, researchers have further analyzed high-affinity sites in non-AD 4R tauopathies. While both core and entry sites exhibit higher binding affinities than surface sites, a key challenge is that the tau tracers may struggle to access these sites within the extended architecture of 4R-tau [33]. Therefore, research on the binding sites of PSP and CBD focuses on the surface sites. The principal surface binding regions are illustrated in Fig. 2b.

Different tau PET tracers display unique binding characteristics at these high-affinity binding sites. For example, Li et al. compared the binding profiles of multiple tracers—including ^18^F-APN-1607 and ^18^F-PI-2620—at the major surface sites in PSP (S1–S7), CBD (s6, s9, and s11), and AD (V1–V4) (Table 1). These surface sites typically contain 2–4 residues. The structural features of the S6 site of PSP and the V2 site of AD are similar, with high docking scores for most tracers (− 7 to − 8 kcal/mol for ^18^F-CBD2115 and ^18^F-APN-1607), and both of these sites are relatively stable according to the molecular dynamics simulations [34].

**Table 1:** PET tracer binding to surface sites of different tau fibrils

Tracer	PSP (4R-tau)	CBD (4R-tau)	AD (3R/4R-tau)
^18^F-APN-1607	Docking Score: − 7.4 kcal/mol (S6) Preferred Sites: S4, S6 Stability (MD): Stable	Docking Score: − 6.3 kcal/mol (s9) Preferred Sites: s9 Stability (MD): Stable	Docking Score: − 7.4 kcal/mol (V2) Preferred Sites: V2, V4 Stability (MD): Stable
^18^F-PI-2620	Docking Score: − 6.8 kcal/mol (S6) Preferred Sites: Q^276^[I^277^]I^278^ Stability (MD): Stable	Docking Score: Not top-ranked Preferred Sites: s11 Stability (MD): Stable	Docking Score: − 6.4 kcal/mol (V2) Preferred Sites: V2, V4 Stability (MD): Stable
Flortaucipir	Docking Score: worse than − 6.0 kcal/mol. Preferred Sites: K^274^[V^275^]Q^276^ Stability (MD): Stable	Docking Score: worse than − 6.0 kcal/mol. Preferred Sites: Contacts R^349^ Stability (MD): Moderate	Docking Score: − 7.2 kcal/mol (V2) Preferred Sites: V2 Stability (MD): Stable
^18^F-CBD-2115	Docking Score: − 7.5 kcal/mol (S6) Preferred Sites: S2, S6 Stability (MD): Stable	Docking Score: − 6.7 kcal/mol (s6) Preferred Sites: Migrates to T^361^[H^362^]V^363^ Stability (MD): Unstable	Docking Score: − 7.7 kcal/mol (V2) Preferred Sites: V1, V2, V4 Stability (MD): Stable

PET: positron emission tomography; PSP: progressive supranuclear palsy; CBD: corticobasal degeneration; AD: Alzheimer’s disease; 4R-tau: four-repeat tau; 3R-tau: three-repeat tau; MD: molecular dynamics. This table summarizes the work of Li et al. [34], which is published under a CC-BY 4.0 license

In the same study, compared with ^18^F-PI-2620, ^18^F-APN-1607 exhibited stronger binding to the PSP-fold and exhibited more favorable binding free energy at most surface sites [34]. ^18^F-APN-1607 binding at major surface sites in PSP is relatively stable during the simulation process [34]. Despite these differences, currently investigated tau PET tracers lack specificity for 4R-tau [33]. In the AD fold, tracers benefit from easier access to entry and core sites, as well as groove-like concave sites that enhance PET imaging. With the elucidation of the near-atomic tau structure, the development of tracers with higher specificity and binding affinity for 4R tauopathies is expected to advance more rapidly [19].

To date, most tau PET tracers target primarily AD-type NFTs with high affinity. However, developing tracers with greater specificity for 4R tau can improve the diagnostic specificity and sensitivity for 4R tauopathies such as PSP [35]. Tau PET tracers, including ^18^F-CBD-2115 (^18^F-OXD-2115), ^18^F-PI-2620 derivatives, and ^18^F-OXD-2314, demonstrate enhanced binding selectivity for 4R tau aggregates, positioning them as promising candidates for diagnosing 4R tauopathies [36, 37]. Leveraging these core-binding ligand structures, contemporary de novo ligand discovery strategies are employing computer-aided methods to screen compound libraries for structural analogs with superior physicochemical properties. Furthermore, cryo-EM is being integrated with a multiscale simulation workflow to characterize tracer-binding modes to tau [38].

## Intracellular distribution of tau

Under physiological conditions, tau is predominantly expressed in the axons of neurons. It mainly functions to stabilize axonal microtubules, thereby mitigating their dynamic instability [14]. Emerging evidence indicates that the *MAPT* gene is also expressed in astrocytes and oligodendrocytes, where the translated tau proteins perform physiological functions [39]. Under pathological conditions, posttranslationally modified tau proteins dissociate from microtubules and primarily accumulate in the somatodendritic domain, leading to synaptic dysfunction. Research suggests that the dendritic spines become major sites of tau-mediated toxicity in postsynaptic compartments [40, 41]. In addition, a smaller fraction of pathological tau species at presynaptic terminals disrupt the presynaptic neurotransmission by impairing vesicle release [42].

The cellular tau distribution in PSP can manifest as NFTs, threads, and globose tangles within neurons, as well as tufted astrocytes and coiled bodies in oligodendroglia. In contrast, tau pathology in AD predominantly affects neurons, with limited glial involvement [11]. Pathological tau is also associated with reduced nuclear tau, impairing the physiological role of tau in preventing DNA damage, thus contributing to genomic instability [43]. Additionally, recent studies highlight that *MAPT* transcripts remain detectable in neurons and glia with tau aggregation. These cells not only internalize misfolded tau species but also maintain endogenous tau production through sustained *MAPT* expression. This dual contribution may generate a persistent pool of tau proteins for phosphorylation and aggregation or fuel the propagation of misfolded tau seeds by providing physiological tau substrates, thereby potentially amplifying the pathological cascade [44].

Glial cells play crucial roles in supporting neuronal function and maintaining the homeostasis of the central nervous system. Astrocytes have long been recognized for their essential role in providing structural, metabolic, and functional support to neuronal networks through their perisynaptic processes, which form a critical component of the tripartite synapses [45–47]. Therefore, the astrocyte dysfunction may play an important role in the pathogenesis of 4R tauopathies [48]. The pathology of tau in astrocytes is associated with cellular reactivity and dysfunction [49]. Different tau species are preferentially internalized by astrocytes, a process that requires amyloid precursor protein and heparan sulfate [50]. Early in disease progression, the astrocytes in PSP and CBD appear to sequester extracellular tau into relatively inert, NFT-like aggregates, whereas synaptic dysfunction tends to emerge at later stages [51, 52].

**Fig. 3:**
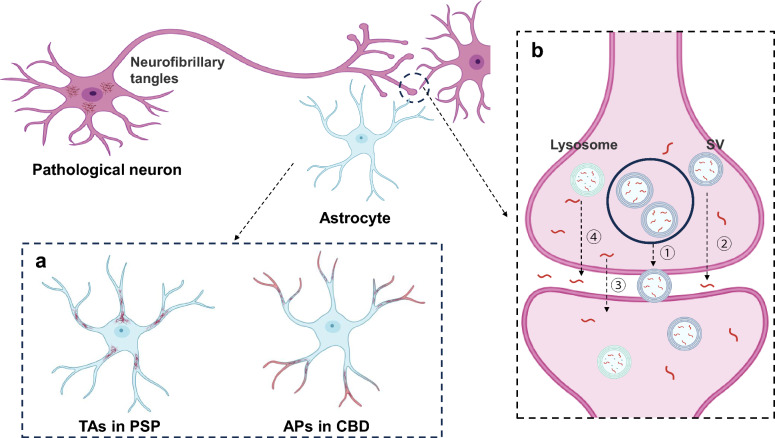
Characteristics of astrocytic tau pathology and interneuronal tau transmission. **a** Distinct patterns of astrocytic tau deposition are observed in progressive supranuclear palsy (PSP) and corticobasal degeneration (CBD), designated tufted astrocytes (TAs) and astrocytic plaques (APs), respectively. These pathological entities demonstrate differential cellular distributions: TAs in PSP tend to localize to cellular regions near the cell body, whereas APs in CBD more frequently accumulate at distal terminals. CBD-associated astrocytic lesions exhibit more pronounced neuropathological consequences at axonal terminals. **b** Potential routes of neuron-to-neuron tau propagation have been proposed to be proximity dependent, including ① extracellular vesicle pathways, ② synaptic vesicle (SV)-mediated transport, ③ direct transmembrane secretion, and ④ secretory lysosome-dependent release. The figure was created with Biorender.com.

Notably, PSP and CBD demonstrate distinct patterns of astrocytic tau pathology. In PSP, the hyperphosphorylated tau in astrocytes is predominantly localized to cellular compartments near the cell body, whereas in CBD, the astrocytic plaques exhibit a more widespread distribution, often appearing far from the cell soma [53, 54]. The functional consequences of these distinct tau distributions are clinically significant. PSP-associated tufted astrocytes are weakly associated with synaptic alterations, whereas CBD-related astrocytic plaques demonstrate a more pronounced disruption of synaptic integrity (Fig. 3a) [55].

## Intercellular spread of tau proteins

Cellular tau can be released into the extracellular matrix physiologically. The concentration of tau in the interstitial fluid resides within the nanomolar range, approximately 1000-fold lower than that in neurons but approximately tenfold higher than that in the cerebrospinal fluid [16]. Under pathophysiological conditions, the majority of extracellularly released tau consists of C-terminally truncated fragmented tau [56]. Tau transmission likely occurs via a proximity-dependent mechanism, inducing further aggregation through conformational changes templated by distinct tau structures [16, 57]. Cellular tau is released mainly through four pathways: extracellular vesicle-mediated secretion, synaptic vesicle shedding, direct translocation across the plasma membrane, and release via secretory lysosomes (Fig. 3b) [58]. Extracellular vesicle-mediated secretion is thought to be the most important pathway for the removal of full-length tau from cells [59]. Most tau in the interstitial fluid is internalized by cells, locally degraded, or drained through the perivascular space.

A critical debate persists regarding the extent to which tau aggregation serves as a seed for the spatial propagation of tauopathies. While the findings of some studies suggest that extracellular tau aggregates can be internalized and exert pathological effects within cells [60], mature tau fibrils are unlikely to function as disease-initiating seeds because their size hinders intercellular transport. Compared with mature fibrils, soluble tau oligomers exhibit markedly greater seeding potency [61]. However, the minimum tau unit capable for seeding remains undefined. Emerging evidence suggests that even misfolded tau monomers may possess seeding ability [62–65].

The differences in cellular distribution and seeding activity of tau may depend primarily on tau conformation and the cellular environment [55, 66, 67]. In one comprehensive study, researchers successfully isolated and characterized 18 distinct tau strains based on biochemical and biological criteria. The different tau strains were subsequently inoculated into PS19 transgenic tau mice, resulting in the induction of strain-specific intracellular pathology in distinct cell types and different rates of network propagation [67]. In another study, pathological tau extracted from postmortem human brains was intracerebrally injected into wild-type mice, and induced the formation and propagation of endogenous mouse tau aggregates. This approach revealed different seeding activities derived from AD-folds, CBD-folds, and PSP-folds. Furthermore, the study revealed cell type specificity, with PSP-folds and CBD-folds inducing tau inclusions in glial cells, whereas AD-folds primarily affected neurons [55]. Comparison of in vivo extracted AD-tau and in vitro synthesized AD-tau revealed distinct tau conformations with different seeding activities. These functional disparities may arise from variations in PTMs and tertiary folding patterns, further underscoring the importance of tau conformation to seeding activity [68, 69].

## Spatial distribution of tau proteins in PSP and difference from other tauopathies

Research on the spatial distribution of tau indicates that while different pathological tau strains can induce neuronal tau aggregation in overlapping brain regions, the initial site of tau deposition and the underlying neuronal connectome largely influence the spread of pathology to specific brain areas [55, 70]. In AD, tau aggregation initiates in the transentorhinal region and propagates along highly interconnected “hub” regions, with strongly connected neural nodes exhibiting greater tau burdens [71]. In contrast, PSP-associated tau pathology initially accumulates in subcortical and brainstem regions with elevated metabolic demand [69]. Additionally, emerging evidence indicates that the glial-mediated tau transmission may contribute to the spatial progression of PSP-tau pathology [55]. Tau deposition in the CBD is typically prominent in the frontoparietal cortex, where astrocytic plaques constitute an early and characteristic pathological feature [72, 73].

Tau pathology in AD often aligns with the Braak staging scheme. It first occurs in the entorhinal cortex (Braak stages I and II), then in the hippocampus (stages III and IV), subsequently spreading throughout the cortex (stages V and VI) [74]. Conversely, the spatial distribution of tau in PSP is characterized by involvement of the Pallido–Nigro–Luysian axis. Then the tau pathology follows two potential patterns of craniocaudal development: one involving rostral transmission to the neocortical regions, and the other involving caudal spread to the cerebellum, including the dentate nucleus (Table 2) [6, 7]. In CBD, the spatial pattern of tau pathology has been delineated largely based on the distribution of astrocytic plaques. These lesions follow a highly stereotyped topographic sequence: they emerge in the frontoparietal cortex, extend to the temporal and occipital cortices, subsequently involve subcortical nuclei, and ultimately reach the brainstem [72, 75].

**Table 2:** Comparison of regional differences between the pathological and ^18^F-APN-1607 PET staging systems proposed for PSP

Pathological staging system proposed by Kovacs et al.		PET staging system proposed by Liu et al.	
Stage	Brain regions	Stage	Brain regions
1–2	GP/STN/STR	I-1, I-2	RN/STN/Ra/GP
		II-1, II-2	SN/LC/PU/TH
3–4	FR/DE/CB	III, IV	DE/CB/OC/PA/TE/FR
5–6	OC	–	–

PET: positron emission tomography; PSP: progressive supranuclear palsy; GP: globus pallidus; STN: subthalamic nucleus; STR: striatum; RN: red nucleus; Ra: Raphe; FR: frontal lobe; DE: dentate nucleus; CB: cerebellar white matter; SN: substantia nigra; LC: locus ceruleus; PU: putamen; TH: thalamus; OC: occipital cortex; PA: parietal cortex; TE, temporal cortex. The data are summarized from References 6 and 83, which are published under a CC-BY 4.0 license

The seminal work by Kovacs and colleagues led to the proposal of a six-stage hierarchical model of tau pathology progression in PSP, incorporating both regional distribution and cellular tropism (Table 2). In the initial disease phase (Stage I), mild-to-moderate tau pathology emerges predominantly in subthalamic nucleus neurons, globus pallidus neurons and/or oligodendrocytes, along with striatal astrocytes. Stage II is characterized by an intensification of pathology in these regions, accompanied by the appearance of isolated cellular tau deposits in frontal cortical areas and/or the dentate nucleus/cerebellum. The intermediate phases (Stages III–IV) involve the development of astrocytic tau pathology in the frontal cortices and/or neuronal tau aggregates in the dentate nucleus, combined with oligodendroglial pathology in cerebellar white matter. The terminal stages (Stages V–VI) demonstrate progressive accumulation of astrocytic tau pathology, which increases from mild to moderate/severe levels in occipital cortical regions [6]. Briggs et al. subsequently refined this pathological staging system through rigorous clinicopathological correlation studies, establishing the association between the PSP staging system and clinical disease severity [76].

## Utility of tau PET imaging in PSP

### Development and diagnostic power of tau PET

First-generation tau PET tracers have shown both diagnostic potential and technical constraints, including off-target binding to α-synuclein and rapid metabolic inactivation in vivo [77, 78]. To overcome these limitations, second-generation tau tracers (e.g., ^18^F-PI-2620 and ^18^F-APN-1607) have been engineered with enhanced specificity.

^18^F-PI-2620, structurally optimized from ^18^F-AV-1451 and ^18^F-RO6958948, shows markedly reduced affinity for monoamine oxidase-A (MAO-A), indicating that it is a critical source of off-target binding [79]. In a multicenter study by Brendel et al., ^18^F-PI-2620 PET in the internal globus pallidus (iGP), external globus pallidus, and subthalamic nucleus discriminated PSP from α-synucleinopathies and AD. Using a region-based z score method with tau positivity threshold set as z score ≥ 2, which is calculated as (individual standardized uptake value ratio (SUVR) − healthy control mean SUVR)/healthy control SD, the iGP achieved the highest diagnostic accuracy. This study reported 85% sensitivity and 77% specificity for distinguishing the PSP-RS group from the other groups. Postmortem autoradiographic validation confirmed these in vivo observations, although there were no significant correlations between regional tracer uptake and disease duration or clinical severity[80].

Concurrently, ^18^F-APN-1607—a propanol-modified derivative of ^11^C-PBB3—was developed with improved metabolic stability and specificity for PSP-associated tau aggregates. In vitro studies have confirmed its selective binding to pathological tau isoforms, which is unaffected by MAO-A/B inhibitors. In addition, the ^18^F labeling enhances clinical practicality [81]. Through multimodal assessments spanning animal models, in vivo imaging, and histopathological correlation, Tagai et al. have validated its translational potential [81]. Li et al. further demonstrated that ^18^F-APN-1607 effectively differentiated PSP patients from patients with α-synucleinopathies (with 85% sensitivity and 71% specificity) and healthy controls, based on tau deposition patterns in the Pallido–Nigro–Luysian axis and brainstem nuclei (red nucleus, raphe nuclei). In addition, the SUVRs measured in these regions correlated significantly with the severity of PSP [82].

### Association of postmortem evidence with tau PET imaging

**Fig. 4:**
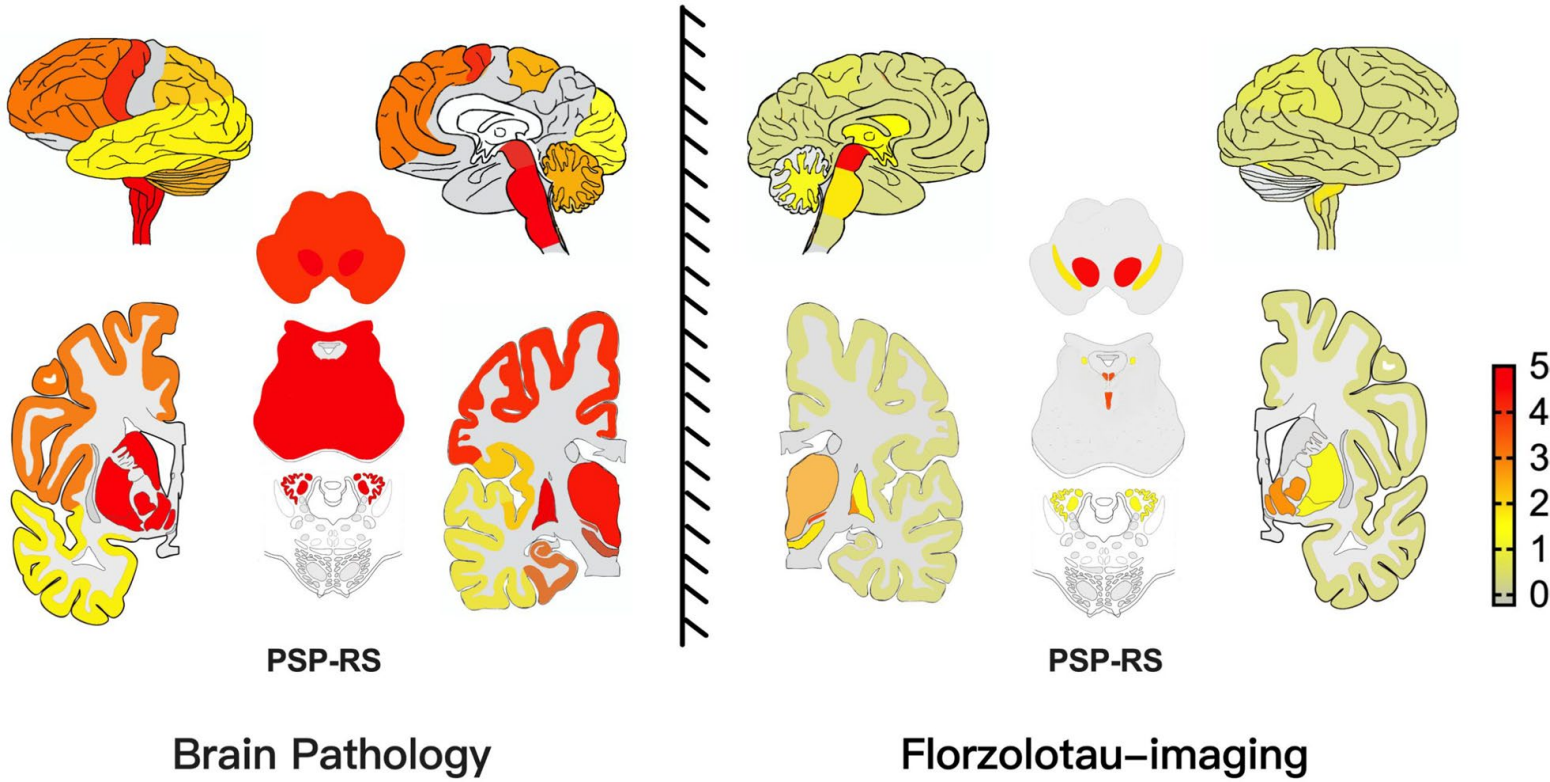
Comparison of tau distribution patterns between postmortem studies and ^18^F-florzolotau (^18^F-APN-1607). Abbreviation: PSP-RS, progressive supranuclear palsy-Richardson’s syndrome. The color bar indicates the SUVR z scores. This figure was reproduced from Reference 83, published under a CC-BY 4.0 license

Liu et al. applied the ^18^F-APN-1607 pathological staging system of PSP in a cohort of 148 patients with PSP, based on the scheme originally developed for post-mortem data. However, they reported a limited correlation between in vivo tau staging and disease severity (Fig. 4). They postulated that the discrepancy might stem from several factors. First, the sample size was insufficient for the advanced disease stages (III–VI), which may cause inherent selection bias, as the post-mortem studies predominantly included cases with prominent tau involvement (stages III–VI) rather than early subcortical tau pathology (stages I–II). Second, the potential limitations in tracer sensitivity for detecting 4R-tau pathology should not be overlooked [83].

Therefore, the researchers further proposed a 4-level staging system for ^18^F-APN-1607 PET imaging in PSP. The staging system categorizes tau pathology progression as follows: Stages I-1 and I-2 (Cluster I) demonstrate initial ^18^F-APN-1607 tracer binding in the red nucleus, subthalamic nucleus, raphe nucleus, and globus pallidus. Subsequent Stages II-1 and II-2 (Cluster II) manifest progressive tracer accumulation in midbrain and diencephalic structures, specifically the substantia nigra, locus coeruleus, putamen, and thalamus. Advanced Stages III–VI (Cluster III) exhibit extensive pathological involvement characterized by tracer binding in the dentate nucleus, cerebellar white matter, and ultimately the cortical regions [83]. This hierarchical classification system highlights the early emergence of tau pathology in the brainstem nuclei, particularly the red nucleus, raphe, locus coeruleus, and substantia nigra, providing a neuroimaging framework for detecting pathological changes during prodromal phases (Table 2).

A pattern divergence between in vivo tau PET imaging and postmortem histopathological studies in PSP has been observed in the cortex (Fig. 4). Kovacs et al. reported that tau-laden astrocytes are predominant contributors to cortical tau pathology in PSP patients [6]. Therefore, tau PET tracers may underestimate the cortical tau load in PSP patients because of the limited affinity of the tracers for astrocytes. In 4R-tau-transgenic mice (PS19), compared with astrocytes, the uptake of ^18^F-PI-2620 is markedly greater in neurons, indicating that tau-positive astrocytes may not be the main contributors to its autoradiographic signal [84]. Additional investigations in PSP brain tissues are needed to elucidate and quantify the binding characteristics of tau PET tracers to astrocytic tau pathology.

Furthermore, potential off-target binding may also contribute to the discrepancies in the pattern of tau pathology between in vivo PET imaging and postmortem studies. Off-target binding of ^18^F-PI-2620 to the meninges may affect signal quantification in regions such as the lateral parietal cortex and the anterior superior cerebellum [85]. In addition, the off-target interactions with neuromelanin, melanin-containing cells, and cerebral microhemorrhages may influence tracer uptake in structures such as the substantia nigra and locus coeruleus [86]. ^18^F-APN-1607 exhibits off-target binding in the choroid plexus, which may confound the interpretation of signals in adjacent regions, including the periventricular areas, the hippocampus, and the thalamus [81]. Recent findings indicate that the prominent ^18^F-APN-1607 uptake in the choroid plexus may be attributed to its binding to the transmembrane protein 106B (TMEM106B) aggregates, which form amyloid filaments in the aging brain [87, 88]. Moreover, the interaction of ^18^F-APN-1607 with Aβ deposits should not be overlooked, as in vitro autoradiography of human brain tissue has demonstrated that ^3^H-APN-1607 shows measurable affinity for Aβ plaques [89].

### Differentiation of PSP subtypes using tau PET

Patients with PSP exhibit significant clinical and pathological heterogeneity, characterized by diverse patterns of tau deposition and disease progression. The potential utility of tau PET imaging in delineating disease subtypes and informing personalized therapeutic strategies remains a critical area of investigation. Initial studies utilizing the ^18^F-PI-2620 tracer revealed no significant differences in regional tau uptake across tau-sensitive brain regions when PSP-RS was compared with other clinical subtypes, including PSP-P, PSP-F, and PSP-CBS. Nonetheless, a graded pattern was observed: PSP-RS showed the highest cumulative z scores across all evaluated regions, followed by PSP-P, PSP-F, and PSP-CBS [80]. In contrast to these findings, Liu et al. employed ^18^F-APN-1607 PET to identify distinct in vivo tau pathology distribution patterns across PSP subtypes. Their analysis of 84 PSP-RS and 64 PSP-non-RS patients (predominantly PSP-P and PSP-PGF) revealed significant between-group differences in subcortical tau deposition [83].

Machine learning algorithms are expected to enhance PSP subtyping through pattern recognition. Hong et al. implemented the subtype and stage inference algorithm in a cohort of 148 clinically diagnosed PSP patients and identified two distinct tau pathology progression patterns based on spatial and temporal features. The Subtype 1 demonstrates sequential tau propagation from subcortical to cortical regions with accelerated regional tau accumulation, which is correlated with more severe clinical manifestations, including cognitive impairment, bulbar dysfunction, limb symptoms, and global motor deterioration. In contrast, the Subtype 2 patients exhibit concurrent subcortical and cortical tau deposition from disease onset, which is associated with slower progression rates and relatively mild symptom profiles [90].

### Advances in tau PET imaging analysis

Multiple studies have reported comparable cortical tau deposition levels between PSP patients and healthy controls, but these observations conflict with the observed cognitive impairments in patients with PSP [80, 83]. Apart from the inherent imaging characteristics of tau tracers, this discrepancy may stem from methodological limitations in tau PET quantification, particularly the reference region selection. Most existing studies adopted AD-derived reference protocols utilizing cerebellar-related regions. However, these reference regions may not be suitable in PSP, as there is neuropathological evidence of early cerebellar tau deposition in PSP, particularly within the dentate nucleus and cerebellar white matter [6, 10, 91, 92]. Therefore, it is necessary to compare the magnitude and variability of the standardized uptake values across different reference regions. Furthermore, by calculating the regional SUVRs with different reference regions, the ability to differentiate disease conditions, as well as the correlations with disease severity, can be assessed [93, 94].

Emerging quantification methodologies show promise in addressing these technical challenges. The parameter estimation for reference signal intensity (PERSI) approach, initially developed for AD studies, employs bimodal Gaussian distribution modeling to identify patient-specific white matter as the reference region. Compared with conventional cerebellar reference regions, this approach demonstrates enhanced sensitivity in PSP applications [93, 95]. Hsu et al. demonstrated that the PERSI-based SUVRs in frontal, parietal, and occipital cortices significantly improved group differentiation between PSP patients and healthy controls. These discriminative capabilities were absent when cerebellar white matter was used as the reference region. In addition, the PERSI-based cortical tau measures correlated significantly with Mini-Mental State Examination scores, suggesting the clinical relevance of this quantification approach [96]. However, this method requires PET and high-resolution 3D MRI acquisition and complex image processing, which limits its clinical translation. Subsequent research should evaluate whether simplified alternatives (e.g., eroded white matter) can achieve comparable performance to that of the PERSI [97].

In addition to direct mapping of tau deposition, complementary techniques, including dynamic perfusion imaging, provide critical insights into the functional consequences of neurodegeneration [98, 99]. Dynamic ^18^F-PI-2620 PET protocols enable the simultaneous assessment of tau load and cerebral perfusion patterns, revealing distinct spatial profiles in patients with PSP. Comparative analyses revealed significant hypoperfusion across 21 of the 246 brain regions in the PSP and CBS cohorts, predominantly affecting the thalamus, caudate, and anterior cingulate cortex. Multivariate modeling of perfusion abnormalities and tau accumulation achieved superior diagnostic differentiation between PSP/CBS and AD/frontotemporal dementia (FTD) (area under the curve: 0.903) and correlated with clinical severity measured by the progressive supranuclear palsy rating scale (*R* = 0.402;* P* = 0.0012) [100]. Subcortical tau deposition in PSP patients is inversely related to cortical perfusion in the limbic and frontal regions, suggesting that tau-mediated disruption of cortico-subcortical connectivity may drive hypoperfusion through functional disconnection mechanisms [101]. This hypothesis is reinforced by template-based analyses of cortico-subcortical functional connectivity. A high subcortical tau burden is spatially correlated with hypoperfusion in functionally connected cortical territories, indicating that tau propagation along neural networks may mediate remote metabolic dysfunction [98].

## Conclusions and future perspectives

In this review, by connecting molecular insights with systems-level neuroimaging, we elucidate the molecular architecture of tau filaments and possible propagation mechanisms, including the cryo-EM structural conformation of tau isoforms, aberrant intraneuronal redistribution, and transsynaptic spreading patterns. Tau PET has shown great potential in clinical utility and enables the in vivo detection of tau. Second-generation tau tracers, including ^18^F-PI-2620 and ^18^F-APN-1607, exhibit enhanced specificity for detecting topographical tau distribution patterns in PSP, enabling improved discrimination of overlapping disorders. Emerging clinical trials demonstrate the utility of tau PET for monitoring disease progression and evaluating therapeutic effects.

Despite significant advances, key challenges remain in the application of tau-PET imaging for PSP. First, the sensitivity of current tracers to early subcortical tau pathology needs to be further evaluated, especially in prodromal stages or PSP-non-RS subtypes. Second, the discrepancy between cortical tau PET signals and cognitive dysfunction may reflect either methodological limitations (e.g., reference region selection) or intrinsic limitations of the current tracers. Third, the extent of off-target binding of second-generation tau tracers to neuromelanin, microhemorrhages, TMEM106B in the choroid plexus, Aβ or other pathological proteins requires careful evaluation.

Addressing these gaps will require the development of tau tracers with higher affinity for 4R-tau, standardized quantification methods accounting for PSP-specific reference regions, and potential off-target binding evaluation. Furthermore, the integration of tau PET with complementary fluid biomarkers and multimodal neuroimaging will assist in the multidimensional diagnosis of PSP with early detection and intervention.

## Data Availability

Not applicable.

## Abbreviations

PET: Positron emission tomography
PSP: Progressive supranuclear palsy
PSP-RS: PSP-Richardson’s syndrome
PSP-P: PSP-parkinsonism
PSP-PGF: PSP-progressive freezing gait
PSP-CBS: PSP-corticobasal syndrome
PSP-F: PSP-frontal syndrome
NFTs: Neurofibrillary tangles
cryo-EM: Cryo-electron microscopy
MTBR: Microtubule-binding region
3R-tau: Three-repeat tau
4R-tau: Four-repeat tau
PTMs: Posttranscriptional modifications
PHFs: Paired helical filaments
SFs: Straight filaments
AD: Alzheimer’s disease
CBD: Corticobasal degeneration
MAO: Monoamine oxidase
iGP: Internal globus pallidus
SUVR: Standardized uptake value ratio
PERSI: Parameter estimation for reference signal intensity
